# A *Clostridium* Group IV Species Dominates and Suppresses a Mixed Culture Fermentation by Tolerance to Medium Chain Fatty Acids Products

**DOI:** 10.3389/fbioe.2017.00008

**Published:** 2017-02-20

**Authors:** Stephen J. Andersen, Vicky De Groof, Way Cern Khor, Hugo Roume, Ruben Props, Marta Coma, Korneel Rabaey

**Affiliations:** ^1^Center for Microbial Ecology and Technology (CMET), Ghent University, Gent, Belgium; ^2^Center for Sustainable Chemical Technologies, University of Bath, Bath, UK

**Keywords:** carboxylic acids, *Clostridium*, fermentation, MCFA, microbial community, toxicity

## Abstract

A microbial community is engaged in a complex economy of cooperation and competition for carbon and energy. In engineered systems such as anaerobic digestion and fermentation, these relationships are exploited for conversion of a broad range of substrates into products, such as biogas, ethanol, and carboxylic acids. Medium chain fatty acids (MCFAs), for example, hexanoic acid, are valuable, energy dense microbial fermentation products, however, MCFA tend to exhibit microbial toxicity to a broad range of microorganisms at low concentrations. Here, we operated continuous mixed population MCFA fermentations on biorefinery thin stillage to investigate the community response associated with the production and toxicity of MCFA. In this study, an uncultured species from the *Clostridium* group IV (related to *Clostridium* sp. BS-1) became enriched in two independent reactors that produced hexanoic acid (up to 8.1 g L^−1^), octanoic acid (up to 3.2 g L^−1^), and trace concentrations of decanoic acid. Decanoic acid is reported here for the first time as a possible product of a *Clostridium* group IV species. Other significant species in the community, *Lactobacillus* spp. and *Acetobacterium* sp., generate intermediates in MCFA production, and their collapse in relative abundance resulted in an overall production decrease. A strong correlation was present between the community composition and both the hexanoic acid concentration (*p* = 0.026) and total volatile fatty acid concentration (*p* = 0.003). MCFA suppressed species related to *Clostridium* sp. CPB-6 and *Lactobacillus* spp. to a greater extent than others. The proportion of the species related to *Clostridium* sp. BS-1 over *Clostridium* sp. CPB-6 had a strong correlation with the concentration of octanoic acid (*p* = 0.003). The dominance of this species and the increase in MCFA resulted in an overall toxic effect on the mixed community, most significantly on the *Lactobacillus* spp., which resulted in a decrease in total hexanoic acid concentration to 32 ± 2% below the steady-state average. As opposed to the current view of MCFA toxicity broadly leading to production collapse, this study demonstrates that varied tolerance to MCFA within the community can lead to the dominance of some species and the suppression of others, which can result in a decreased productivity of the fermentation.

## Introduction

The conversion of a complex substrate in anaerobic digestion and fermentation relies on the competition for carbon and energy within a mixed microbial community to drive the conversion of diverse polymeric biomass to simpler end products, such as methane, acetic acid, and other volatile fatty acids (VFAs). The environmental conditions in such systems are engineered (pH, temperature, organic loading, and wash out rate) in an attempt to optimize performance throughout this collaborative biological economy. Conditions cannot be ideal for each player, and some applied or inherent conditions can result in favorable (or unfavorable) conditions for some species to the detriment (or benefit) of others, thus impacting the role they play in this process. Managing this principle is at the center of engineering a mixed microbial biotechnology (Angenent et al., 2016; Lindemann et al., 2016).

VFAs are bulk chemicals that can be produced in a biorefinery context from organic waste streams. This can include short chain fatty acids such as acetic and propionic acid, common biological intermediates such as lactic acid and succinic acid, and medium chain fatty acids (MCFAs) such as hexanoic and octanoic acid. Chain elongation of short chain VFA occurs through reverse β-oxidation, in which some species can gain energy by increasing the chain length of VFA with reductive substrates such as ethanol and lactic acid (Seerdorf et al., 2007; Spirito et al., 2014; Angenent et al., 2016). Hexanoic acid has come in focus as a target of bioproduction as it can be used in applications as diverse as antimicrobial animal feed additives and jet fuel precursors. Bioproduction has recently been demonstrated at the laboratory scale at industrially interesting rates (Grootscholten et al., 2013; Ge et al., 2015). Sustainable MCFA production technologies are yet to have reached an industrial scale, but research is on-going and scale-up is underway. Research toward the production of MCFA generally focuses on characterizing the productive species and designing a system based around its preferred conditions and substrates. A body of work exists on *Clostridium kluyveri, Megasphaera elsdenii*, and other species (Seerdorf et al., 2007; Jeon et al., 2010; Choi et al., 2013; Weimer and Moen, 2013; Ge et al., 2015; Angenent et al., 2016; Kucek et al., 2016), and recently a new genus *Caproiciproducens* was proposed to house the isolate *Clostridum* sp. BS-1, under the proposed name of *Caproiciproducens galactitolivorans* (Kim et al., 2015). The *Clostridium* group IV has been shown to generate hexanoic acid from lactic acid (Xu et al., 2015).

MCFA have a pH-dependent microbial toxicity that can disrupt membrane integrity at around 40 mM (4.6 g L^−1^) for hexanoic acid (pK_a_ 4.85) and 20 mM (2.9 g L^−1^) for octanoic acid (pK_a_ 4.89), as characterized in *Escherichia coli* at pH 4.3, with no toxicity demonstrated at pH 7 (Yang et al., 2010; Royce et al., 2013). MCFA fermentation is often performed under acidic conditions to suppress methanogen activity. Stable, continuous operation of a reactor in the presence of MCFA under acidic conditions requires this toxicity to be managed by attentive organic loading, pH compromise and hydraulic residence time, or extraction by a robust *in situ* recovery technology that can work effectively at low concentrations to protect the microbial community. Extraction efficiency and unit operation cost are inherently linked to the concentration of the target compound, and as such limited extraction efficiency and high recovery cost are recognized as major practical and economic hurdles in bringing sustainable MCFA production to an industrial reality (Agler et al., 2011; López-Garzón and Straathof, 2014; Andersen et al., 2015; Xu et al., 2015; Angenent et al., 2016).

The supporting microbial community in an MCFA fermentation from a complex substrate is core to the carbon economy. A single-stage production from waste requires microorganisms to perform hydrolysis, acidogenesis, and as necessary, other fermentations (e.g., toward ethanol and lactic acid) (Agler et al., 2011, 2012; Angenent et al., 2016), while also contributing some critical coproduction of gases (e.g., hydrogen gas and carbon dioxide gas). The supporting community in anaerobic digestion includes the *Bacteroidetes* and *Firmicutes* phyla, which enrich when switching from biogas to VFA production (De Vrieze et al., 2015). *Bacteroidetes* have been related to parallel reactions when *Clostridium* sp. was enriched for MCFA production (Coma et al., 2016). In mixed microbial chain elongation to MCFA, *Lactobacillus* spp. have also been found in communities alongside *Megasphaerea* sp. (Andersen et al., 2015) and *Clostridium* group IV (Zhu et al., 2015), suggesting a lactic acid chain elongation route. Ethanol is often added in studies for mixed culture chain elongation but can also be generated by the supporting community from sugars or acetic acid and carbon dioxide (Spirito et al., 2014). It is arguably somewhat reductive to refer to producers of critical intermediates as a “supporting” community, particularly if the complete utilization of a substrate is critical to the production economics. Within the carbon and energy economy of the reactor microbiome the non-elongating, supporting community is process critical and therefore important to study, understand, and engineer.

Toxicity in an MCFA fermentation, particularly in mixed cultures, is generalized as the broad suppression of microorganisms, leading to the collapse of production. MCFA toxicity is only observed incidentally and, to our knowledge, has not been studied with a mixed culture. This study explores community tolerance and toxicity to MCFA, and by extension the dominance of some MCFA tolerant species and the suppression of others that succumb to the toxicity. In this work, we therefore aimed to describe the microbial relationships in a mixed community MCFA fermentation and specifically investigated how these toxic products impact the community and the process. We describe the operation of two identical reactors fed with biorefinery beer and stillage, an organics-rich stream from the bottoms of a bioethanol distillation column and track changes in the substrate consumption, VFA concentration, and bacterial community composition according to relative abundance.

## Materials and Methods

### Bioreactor Operation

Fermentation of beer and stillage from a wheat bioethanol process (supplied by Tereos Starch & Sweeteners, Aalst, Belgium) was performed in two identical DOLLY twin Bioreactors (Belach Bioteknik AB, Sweden). The working volume was fixed at 5 L and fed at daily intervals from a source stored at 4°C and pH 3.5 ± 0.1. The reactors were maintained at a hydraulic residence time of 7.5 days and pH 5.5, controlled by dosing 5 M NaOH. At start-up, reactors were filled with 4.5 L adapted DSMZ medium 52 and 0.5 L of an enriched, in-house inoculum for MCFA production. After 24 h, 1.75 L of medium were replaced with a 5:2 beer to stillage ratio mixture to target 10 g L^−1^ ethanol in the total reactor volume, followed by regular HRT feeding at approximately 14% beer to target an ethanol concentration of 6 g L^−1^. The feed had a measured 57 ± 9 g COD_Soluble_ L^−1^ and 101 ± 18 g COD_Total_ L^−1^. The concentration of soluble substrates measured included sugars at 9.0 ± 0.4 g L^−1^ of xylose, 4.9 ± 0.2 g L^−1^ arabinose and 2.2 ± 0.1 g L^−1^ glucose (*n* = 4) and 6.0 ± 2.4 g L^−1^ ethanol, 3.8 ± 1.2 g L^−1^ glycerol, 1.6 ± 1.3 g L^−1^ lactic acid, and 0.4 ± 0.2 g L^−1^ acetic acid, at pH 3.5 ± 0.1 and 6.0 ± 0.9 ms cm^−1^ (*n* = 18).

### Sample Analysis

Reactors were sampled three times per week and analyzed for C2 to C8 fatty acids (including isoforms C4 to C6) by gas chromatography (GC-2014, Shimadzu^®^, The Netherlands), with a DB-FFAP 123-3232 column (30 m × 0.32 mm × 0.25 µm; Agilent, Belgium) and a flame ionization detector (FID). Liquid samples were conditioned with 2 mL sulfuric acid, 200 mg sodium chloride, and 2-methyl hexanoic acid as an internal standard for quantification before further extraction with diethyl ether (1:1 volume sample:ether). The sample (1 µL) was injected at 250°C with a split ratio of 50 and a purge flow of 3 mL min^−1^. The oven temperature increased by 10°C min^−1^ from 110 to 250°C where it was maintained for 5 min. The FID had a temperature of 300°C. Nitrogen carrier gas was maintained at a flow rate of 2.49 mL min^−1^. “Total VFA” reported in this study is the quantitative sum of all detected linear, unsaturated carboxylic acids, including MCFA. Decanoic acid (C10) was analyzed by the same method and detected at up to 232 ppm, but interference was observed from unidentified compounds already present in the feed, likely to be lipids and lipid fragments. For a comparative analysis of C10, peaks were normalized between 0 and 1, with the average integration of the C10 peak measured in the feed set at 0 (σ = 7 × 10^−4^, *n* = 4), and the maximum peak set at 1. Normalized C10 integrated peaks were clustered into two discrete groupings. Those clustered close to zero are considered a negative detection of the C10 MCFA product, with an average of −0.03 ± 0.2 (*n* = 6), and those outside with an average of 0.60 ± 0.23 (*n* = 6) considered a positive detection. The separate clusters were compared for distinctness by *k*-means clustering and compared against C8 concentration, *Clostridium* sp. BS-1_sec_ relative abundance, and the ratio of the relative abundance of *Clostridium* sp. BS-1_sec_ to *Clostridium* sp. CPB-6.

The concentrations of lactic acid, formic acid, glycerol, 1,3-propanediol, 1,2-propanediol, methanol, ethanol, propanol, and butanol were analyzed with a 930 Compact IC Flex (Metrohm, Switzerland) ion chromatography system with inline bicarbonate removal (MCS), equipped with an organic acids column (Metrosep 250/7.8; Metrohm) and a guard column cartridge (Metrosep Dual 4/4.6; Metrohm) with a 850 IC conductivity detector. Oven temperature was set at 35°C. A 1 mM H_2_SO_4_ solution was used as eluent at a flow rate of 0.5 mL min^−1^. The concentration of the lowest standard, determining lower limit of quantification, was 1 mg L^−1^. The concentrations of glycerol, 1,3-propanediol, 1,2-propanediol, ethanol and 2-propanol were analyzed with a 930 Compact IC Flex (Metrohm) ion chromatography system, equipped with an alcohols column (Metrosep Carb 2 250/4.0; Metrohm) and a guard column cartrige (Metrosep Trap 1 100/4.0; Metrohm) with an IC amperometric detector. Oven temperature was set at 35°C. A 20 mM NaOH was used as eluent at a flow rate of 0.8 mL min^−1^. The concentration of the lowest standard used, therefore determining lower limit of quantification, was 0.5 mg L^−1^ for glycerol, 1 mg L^−1^ for 1,2-propanediol and 2-propanol, and 5 mg L^−1^ for 1,3-propanediol and ethanol. The gas-phase composition was analyzed with a Compact GC (Global Analyser Solutions, Breda, The Netherlands), equipped with a Molsieve 5A precolumn and Porabond column (CH_4_, O_2_, H_2_, and N_2_) and a Rt-Q-bond precolumn and column (CO_2_, N_2_O, and H_2_S). Gases in the headspace were determined by a thermal conductivity detector. COD_Total_ was determined according to Standard Methods (APHA, 2005) using the dichromate oxidation method, while COD_Soluble_ was analyzed with Nanocolor^®^ kits (Macherey-Nagel, Germany) after sample filtration at 0.45 µm. Sugars were analyzed by the NREL procedure according to Sluiter et al. (2006), measured with high-performance liquid chromatography (Agilent Varian ProStar 220 SDM, USA; 5 mM H_2_SO_4_ mobile phase, 0.6 mL min^−1^ and 60°C column temperature with a refractive index detector and Rezex H+ column; Aminex).

### Community Analysis

DNA samples for community analysis of each reactor broth and feed were collected on day 0 (inoculum), days 9, 22, 34, 41, 48, 55, and 64 and were centrifuged in 2 mL sterile Micrewtubes^®^ (Simport, Canada). The supernatant was removed, and the samples were stored at −21°C. Following DNA extraction using PowerSoil DNA kit (MoBio), 16S rRNA sequences were amplified and sequenced using Illumina sequencing technology as described by Andersen et al. (2015). Bioinformatic analysis of sequences was executed using the Mothur pipeline as described by Andersen et al. (2015). Three samples were chosen for Sanger sequencing of 16S rRNA by LGC genomics (Berlin, Germany) to identify enriched cultures on the 16S level. These samples were chosen due to their high relative abundance of the uncultured species *Clostridium* sp. BS-1_sec_, with a relative abundance of approximately 80% from days 34 and 41 from Reactor 1, and day 62 from Reactor 2. Only day 34 from Reactor 1 returned a valid sequence. The alignment considered 974 bp of the phylogenetic gene maker 16S rRNA. The alignment file was submitted to a phylogenetic analysis using the Phylogeny.fr customized workflow service1 including alignment curation with Gblocks2 (using default parameters), tree construction with PhyML3 (using default parameters), and visualization by TreeDyn4 (Castresana, 2000; Chevenet et al., 2006; Dereeper et al., 2008; Guindon et al., 2010). Consensus sequences of all OTUs are available in supplementary information, and raw sequence data are available from the European Nucleotide Archive (ENA) under study accession number PRJEB19351.

### Statistics

Multivariate abundance analysis was used for statistical inference on the relationship between relative species abundances and VFA. All analyses were conducted in R (v3.2.5) with seed 777 using the *mvabund* package (Wang et al., 2012). Samples were rarefied until the minimum sample depth (9,848 reads) and pruned from operational taxonomic units (OTUs), which had a maximum relative abundance lower than 10% or were absent in at least 50% of the samples to specifically focus on the dominant bacterial species. A forward selection-based modeling approach was used. The mean–variance relationship was modeled by a negative binomial distribution, and all models were verified for accordance with the model assumptions as described by Wang et al. (2012). Hypothesis testing was performed using likelihood ratio tests with pit resampling (5,000 runs). The final model consisted of hexanoic acid and total VFA concentration as continuous predictors and the reactor replicate as categorical predictor. Inference on the model parameters of individual species was executed using the adjusted *p*-values, calculated after 5,000 resampling runs to account for family-wise error rates and intervariable correlations. Ordination plots were made using the decorana and rda functions available in the *vegan* package (Oksanen et al., 2015). Detrended correspondence analysis (decorana) was applied on the rarefied community data to avoid horseshoe artifacts that are frequently associated with time series community data. Principal coordinate analysis was used on the VFA dataset.

## Results

### Production of MCFAs and the Collapse of Glycerol Consumption

**Figure 1:**
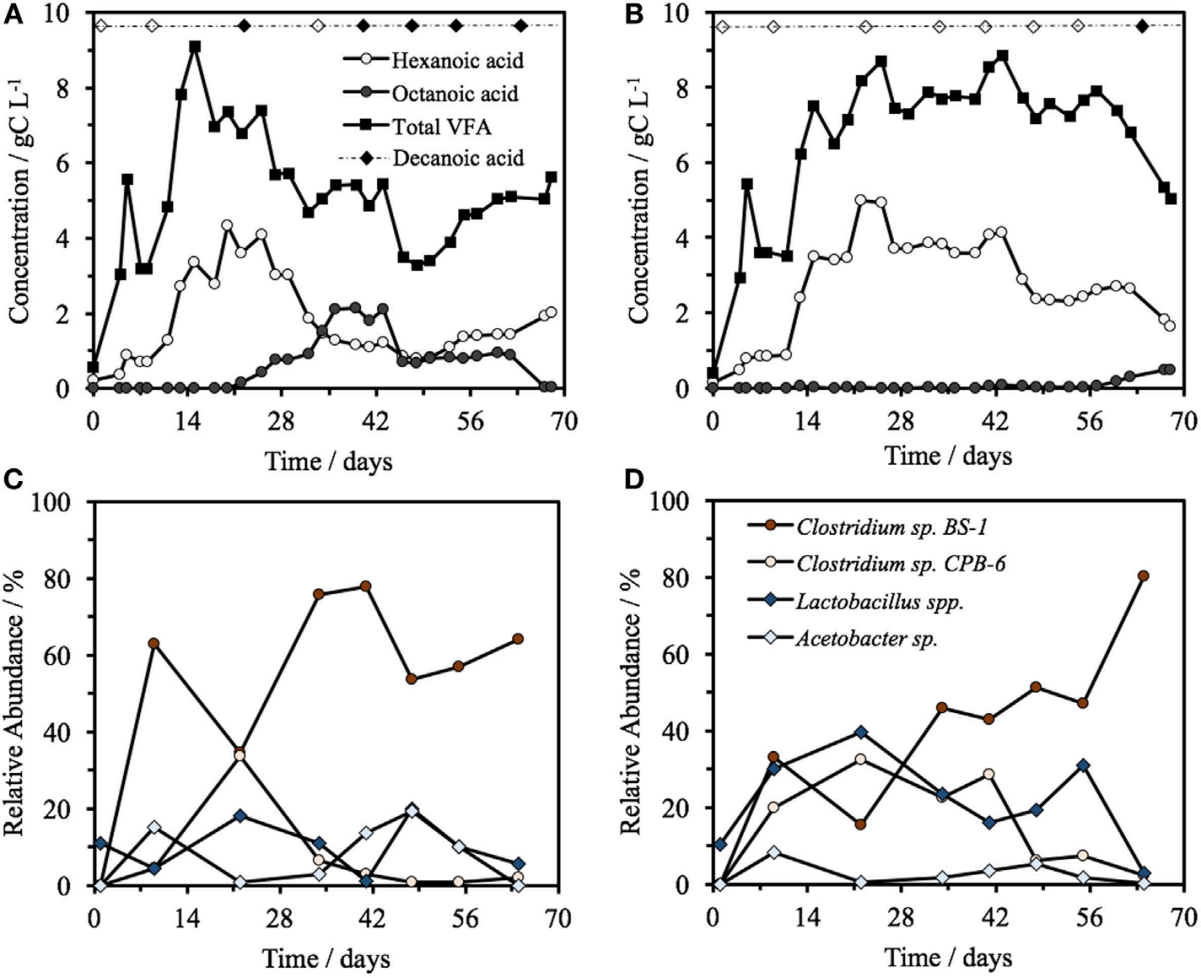
**(A,B)** Concentration over time of total VFA, hexanoic acid, and octanoic acid in Reactor 1 **(A)** and Reactor 2 **(B)** fed from a common source of biorefinery stillage, including the detection of decanoic acid. The filled diamond represents the days at which decanoic acid was detected significantly above the feed baseline, with a blank diamond representing no significant detection. The top four most relatively abundant microorganisms over time in the two identical reactors, Reactor 1 **(C)** and Reactor 2 **(D)**.

Hexanoic acid was consistently produced in both bioreactors at pH 5.5, with a steady-state concentration of 2.0 ± 1.1 gC L^−1^ (3.2 ± 1.8 g L^−1^) in Reactor 1 and 3.2 ± 0.9 gC L^−1^ (5.2 ± 1.5 g L^−1^) in Reactor 2 (Figure 1), including a maximum concentration of 5.0 gC L^−1^ (8.0 g L^−1^) hexanoic acid on day 22 in Reactor 2. As a proportion of the total VFA on a carbon basis, 54 ± 10% of the VFA present was hexanoic, heptanoic, or octanoic acid in Reactor 1 and 45 ± 10% in Reactor 2 (all averages are based on the steady-state period of days 15–68, *n* = 13). The VFA profile of the identical reactors began to diverge at day 20, with Reactor 1 progressively increasing to a maximum of 2.1 gC L^−1^ of octanoic acid by day 39. The total VFA concentration decreased from 7.35 gC L^−1^ on day 20 to a low of 3.46 gC L^−1^ by day 46, 36% lower than the average steady-state concentration. In Reactor 1, this decrease coincided with a change in glycerol consumption. Before day 20, there was an almost complete consumption of glycerol, which shifted to zero net change after day 20, i.e., glycerol detected in the feed was equal to glycerol concentration in the effluent. Reactor 2, operated identically and fed in parallel from the same source, did not show this trend at this time, but rather maintained a total concentration of 7.7 ± 0.6 gC L^−1^ for the days 15–57 with a hexanoic acid concentration of 3.5 ± 0.8 gC L^−1^, and a heptanoic and octanoic concentration between 0 and 0.2 gC L^−1^. The octanoic acid concentration began to rise in Reactor 2 after day 60, which coincided with a decline in both hexanoic acid and the total VFA concentration from 7.4 gC L^−1^ on day 60 to a low of 5.0 gC L^−1^ by day 68, 32% less than the total average steady-state VFA concentration. Decanoic acid was detected in Reactor 1 on days 22 and 41–64 with a relative peak average of 0.50 ± 0.01 (*n* = 6), significantly higher than the baseline of the feed at 0 ± 7 × 10^−4^ (*n* = 4). Decanoic acid was not detected significantly above the baseline in Reactor 2 with a relative peak average of 0.05 ± 2 × 10^−3^ (*n* = 6), with the exception for day 67. Across all samples, decanoic acid detection occurs only when octanoic acid is detected (Figure 1), generally when the concentration of octanoic acid is above around 1 gC L^−1^ (Figure 1; Figure S1 in Supplementary Material).

### Community Domination by Tolerance to MCFA

In both reactors, the community was dominated by *Clostridium* and *Lactobacillus* species (Figures 1C,D). Multivariate abundance testing shows that the community composition was significantly associated with the hexanoic acid concentration (*p* = 0.026) and total VFA concentration (*p* = 0.003) for the community of both reactors, with a significantly different community composition between the two identical reactors (*p* = 0.05). These overall differences could be significantly attributed to the *Lactobacillus* spp. (Otu0001) (*p* = 0.002), which generate lactic acid that the *Clostridia* species are likely to metabolize (Jeon et al., 2010; Kim et al., 2015; Kucek et al., 2016). The two reactors had a high functional similarity in terms of VFA output (Figure S5A in Supplementary Material) despite a large dissimilarity in community profile throughout the 70 days of operation (Figure S5B in Supplementary Material). The peak of dissimilarity in functionality corresponded to the increase of octanoic acid in Reactor 1, and the functional dissimilarity begins to fall as the concentration of octanoic acid in Reactor 2 begins to rise.

**Figure 2:**
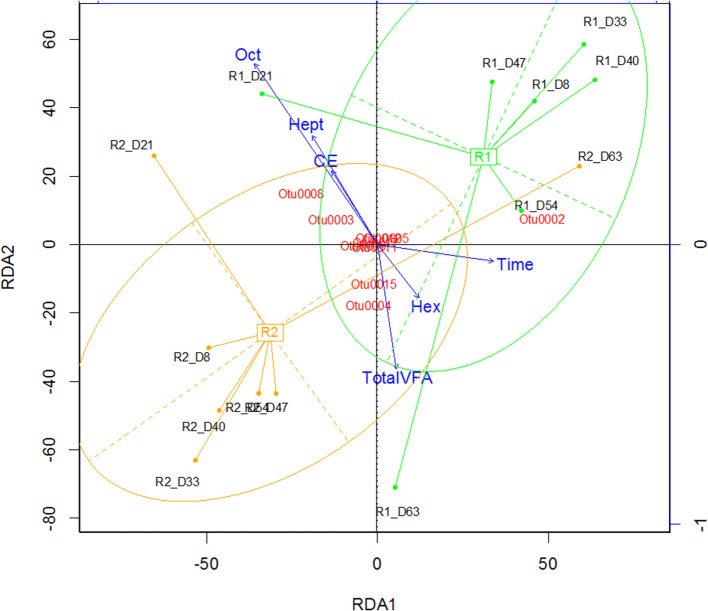
**Redundancy analysis demonstrating the alignment of octanoic acid (Oct), heptanoic acid (Hep), and CE (chain elongation, i.e., concentration of volatile fatty acids greater than C5 chain length) in the opposite direction to the total VFA and hexanoic acid (Hex) concentration**. R*x*_D*y* corresponds to Reactor *x* on day *y*.

The two reactors were dominated by an uncultured *Clostridium* species (Otu004) with 95% similarity to *Clostridium* sp. BS-1 over 427 bp (Table S2 in Supplementary Material). This OTU correlates with the concentration of hexanoic acid and total VFA (Figure 2), and the relative abundance of this species coincides with periods of high octanoic acid and the detection of decanoic acid in both reactors (Figure 1; Figure S2 in Supplementary Material). This species will be referred to as *Clostridium* sp. BS-1_sec_ (i.e., secundum) for brevity, as although it is a relation to *Clostridium* sp. BS-1, the results do not conclusively identify this strain as such. *Clostridium* sp. BS-1 was recently identified as a d-galactitol consuming, fermentive microorganism capable of producing acetic acid, butyric acid, and hexanoic acid (Jeon et al., 2010). Another highly abundant OTU was identified as *Clostridium* sp. CPB-6 (Otu008) with 100% similarity over 427 bp, and this OTU showed a correlation with heptanoic and octanoic acid and high total chain elongation (i.e., the cumulative total concentration of VFA greater than C5 chain length) (Figure 2). As yet, no further information exists on the *Clostridium* sp. CPB-6 strain, although considering it is closely related to *Clostridium* sp. BS-1 and likely a member of the *Clostridium* group IV (96% similarity identified in Otu022), this species may also be able to produce MCFA through chain elongation. *Clostridium* sp. BS-1_sec_ had the greatest relative abundance in both reactors with the exception of day 20 in Reactor 2. In the most stable period of hexanoic production, Reactor 2 had an average *Clostridium* sp. BS-1_sec_ relative abundance between 15 and 46%, the relative abundance of *Clostridium* sp. CPB-6 was between 22 and 32%, and *Lactobacillus* spp. between 16 and 40%. *Lactobacillus* spp. are fermentive microorganisms than can convert a wide variety of substrates into lactic acid, including glycerol (Cantoni and Molnar, 1967). Lactic acid can be used by some microorganisms, including some species in the *Clostridium* group IV (Zhu et al., 2015) to elongate the VFA chain from acetic acid (C2) through butyric acid (C4) to hexanoic acid and longer. Some *Lactobacillus* are able to degrade lactic acid into acetic acid, particularly if oxygen is available as the electron acceptor (Quatravaux et al., 2006). *Acetobacter* sp., present in both reactors, is an obligate aerobe that is able to convert ethanol into acetic acid in the presence of oxygen (Cleenwerck et al., 2002). The reactors were fed from an identical source, an undiluted mixture of biorefinery beer and stillage that was not stored anaerobically, which could account for the abundance and survival of this bacteria. The feed had an average (*n* = 6) relative abundance of 72 ± 19% for *Lactobacillus* spp., 22 ± 19% for *Acetobacter* sp., 3 ± 5% for *Gluconobacter* sp., and 3 ± 1% for other species.

### The Uncultured Species Related to the *Clostridium* Group IV

**Figure 3:**
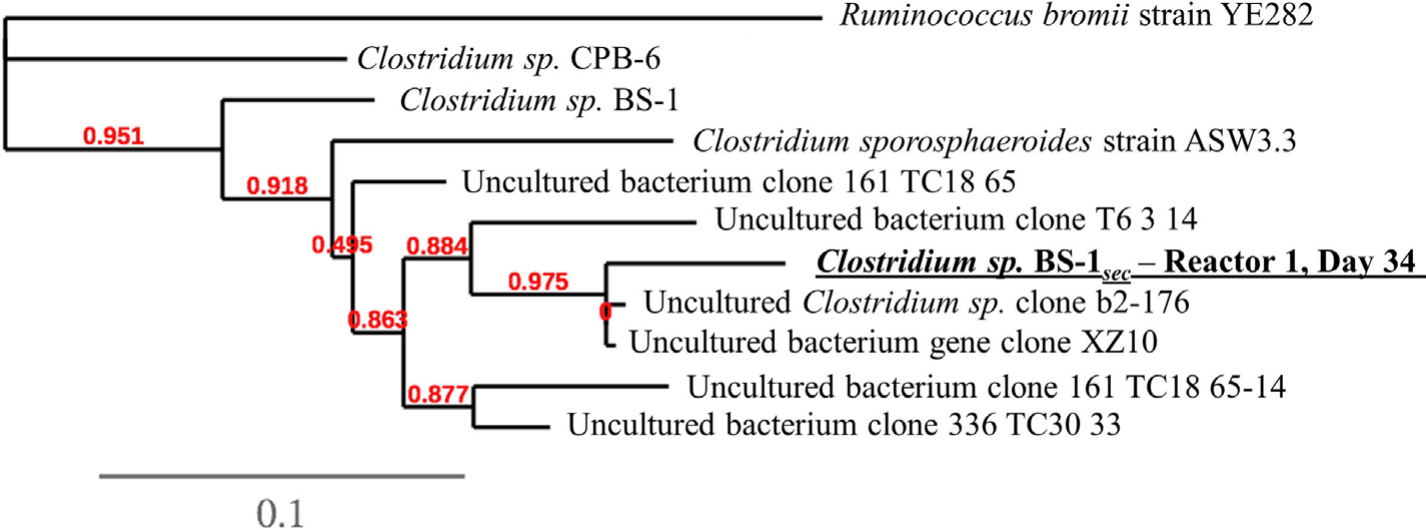
**Phylogenetic tree at species-level resolution showing some genetic heterogeneity among close relatives of the dominant sequence of Reactor 1 on day 34, referred to as *Clostridium* sp. BS-1_sec_**. The scale bar indicates 0.1 estimated changes per nucleotide. The alignment considers 974 bp of the phylogenetic gene maker16S rRNA. See Table S1 in Supplementary Material for more information.

A phylogenetic tree analysis (Figure 3) of the *Clostridium* species referred to as *Clostridium* sp. BS-1_sec_ was generated through long 16S rRNA sequencing based on a sample from Reactor 1 on day 34 in which its relative abundance was 76% (Figure 1). *Clostridium* sp. BS-1_sec_ has a 95% maximum similarity with *Clostridium* sp. BS-1 according to the NCBI BLAST database, with a close relationship to *Clostridium* sp. CPB-6. Both of these *Clostridia* appear to be of the *Clostridium* group IV, recognized as butyrate producers in the gut microbiome (Lopetuso et al., 2013) and capable of elongation of short chain VFA through lactic acid to hexanoic acid (Zhu et al., 2015).

## Discussion

### MCFA Toxicity Decreases Hexanoic Acid Production by Suppressing the Supporting Community

One must take care when implying function from relative abundance, however, some inferences can be made by correlating the VFA products with relative abundance, combined with functional knowledge from previous studies. *Clostridium* sp. BS-1_sec_ is the only species identified with a high relative abundance that is recognized to produce hexanoic acid, likely through the reverse β-oxidation pathway. Reverse β-oxidation can in principal generate octanoic acid and decanoic acid, though decanoic acid has not been previously reported as a product of the reverse β-oxidation pathway. As a close relative, *Clostridium* sp. CPB-6 is likely to also produce hexanoic acid, but there appears to be an antagonistic relationship between the two species. There is a significant linear correlation between the concentration of octanoic acid and the ratio of *Clostridium* sp. BS-1_sec_ to *Clostridium* sp. CPB-6 (*R*^2^ = 0.63, *p* = 0.003, *n* = 12) and a weaker but still significant correlation for the peaks of decanoic acid (*R*^2^ = 0.25, *p* = 0.006, *n* = 12). When both species have similar proportions, the concentration of octanoic acid is low, however, the further *Clostridium* sp. BS-1_sec_ exceeds *Clostridium* sp. CPB-6 in relative abundance, the greater the concentration of octanoic acid. This may be a result of MCFA toxicity decreasing the relative abundance of *Clostridium* sp. CPB-6 or the capacity of *Clostridium* sp. BS-1_sec_ to thrive by generating MCFA through the reverse β-oxidation pathway. Octanoic acid has been reported as product of *C. kluyveri* in mixed cultures (Steinbusch et al., 2011; Spirito et al., 2014), however not explicitly confirmed for any species in pure culture studies. Basic *k*-means clustering the samples (Figures S1, S2, and S4 in Supplementary Material) distinctly separates the negative C10 detection case from the positive case, with the positive C10 peak clusters cooccurring with high concentrations of C8, high relative abundance of *Clostridium* sp. BS-1_sec_, and a high ratio of *Clostridium* sp. BS-1_sec_ to *Clostridium* sp. CPB-6. This supports the hypothesis that the domination of *Clostridium* sp. BS-1_sec_ leads to C8 and C10 MCFA, but here we cannot conclusively state that C10 is a result of reverse β-oxidation production, as opposed to lipid fragments from dead cells (such as yeast or *Lactobacillus* spp.) overlapping the C10 detection.

**Figure 4:**
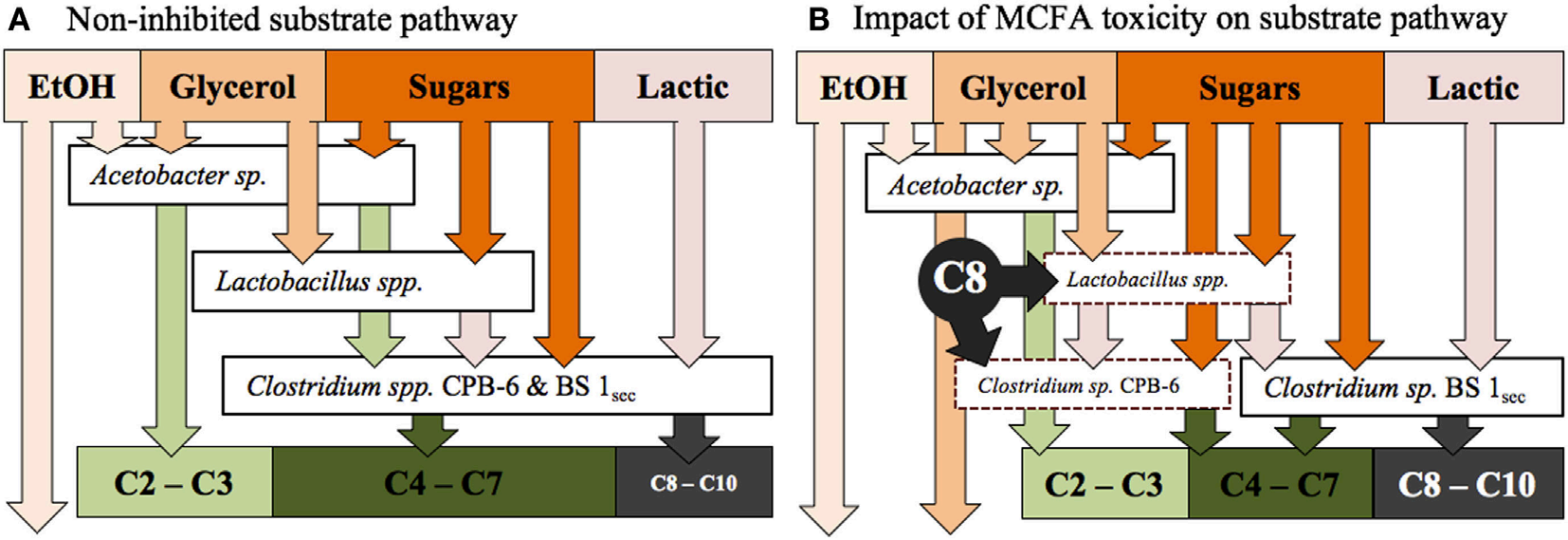
**(A)** The proposed substrate pathway for the fermentation prior to the onset of MCFA toxicity, as observed days 14–21 in Reactor 1 and days 14–52 in Reactor 2. **(B)** The hypothetical impact of MCFA toxicity on the proposed substrate pathway, as observed from days 21 to 68 in Reactor 1. The dashed line borders represent stressed, low relative abundance species.

*Clostridium* sp. BS-1_sec_ is clearly tolerant to MCFA as the only species that increases in relative abundance during moments of high hexanoic and octanoic acid concentration, up to a maximum relative abundance of 78% in Reactor 1 and 80% in Reactor 2. Some species within the *Clostridium* group IV have been observed to remain productive up to a hexanoic acid concentration of 23.4 g L^−1^ at pH 6–6.5, accounting for between 0.5 and 1.5 g L^−1^ of undissociated acid (Zhu et al., 2015). Considering the pK_a_ of hexanoic acid, this is in the same vicinity as the upper limit of 0.87 g L^−1^ of protonated hexanoic acid reported by Angenent et al. (2016). Here, with operation at pH 5.5, one expects a protonated hexanoic and octanoic acid proportion of around 18–20% or an average steady-state concentration of 0.6 ± 0.3 g L^−1^ protonated hexanoic acid in Reactor 1 and 1.0 ± 0.3 g L^−1^ in Reactor 2, with the maximum in Reactor 2 reaching 1.46 g L^−1^. The maximum concentration of undissociated octanoic acid across both reactors is approximately 0.2 g L^−1^, and even at these low concentrations, the supporting community seem to be rather sensitive to this acid. In Reactor 1, *Clostridium* sp. CPB-6 reached a relative abundance of 34% by day 20 and then fell to between 1 and 7% when the octanoic acid concentration rose in the broth. During this period, the relative abundance of *Lactobacillus* spp. shifted between 1 and 20%, with the relative abundance minimum associated with the octanoic acid concentration maximum. In Reactor 2, as *Clostridium* sp. CPB-6 decreased, *Acetobacter* sp. increased in relative abundance to between 3 and 20% from days 20 to 55, before decreasing to less than 1% by the end of the experiment when octanoic acid was at its greatest concentration in this reactor. The relative abundance of *Clostridium* sp. BS-1_sec_ rose to a maximum of 80% in Reactor 2 at this octanoic acid maximum, with the relative abundance of *Clostridium* sp. CPB-6 at 2% and *Lactobacillus* spp. at 3%. The *Lactobacillus* spp. Otu0001 was identified as having a statistically significant association with the total VFA concentration (*p* = 0.0016), which indicates that it plays a significant role in carbon conversion. As mentioned, in Reactor 1, there was a near-total consumption of glycerol until day 25, followed by a shift to almost zero as the octanoic acid concentration rises. This is an indication that the species responsible for glycerol consumption, likely to be *Lactobacillus* spp., were suppressed by octanoic acid and other MCFA (illustrated in Figure 4).

### The Divergence of Identical Reactors

The two identical reactors fed from a common source diverged in the community according to relative abundances, suggesting a rather stochastic community response under the highly complex feed and diverse mixed microbial population. The onset of octanoic and decanoic acid products cannot necessarily be linked to a discrete event in the reactor, however, the variability of the community and the abundance of MCFA producers presented conditions suitable for a “runaway reaction”—a decidedly deterministic toxicity of the supporting community, which drove the *Clostridium* sp. BS-1_sec_ to dominance. The two reactors tended toward a low functional dissimilarity, despite the consistently high dissimilarity of the community. The ability of the *Clostridium* group IV species to take advantage of the short chain VFA end products of other organisms through chain elongation gives them this advantage to runaway to dominance and puts the supporting community at risk of the MCFA products.

Analogous systems in anaerobic digestion and other environmental ecologies often show a high degree of stochastic community turnover prior to a significant environmental disturbance (Zhou et al., 2013; Meerburg et al., 2016), while MCFA fermentation in a mixed community has been shown to be shaped non-randomly in a system with an *in situ* liquid/liquid extraction system, specifically targeting the hydrophobic MCFA (Agler et al., 2012). The toxicity of MCFA on a mixed community, as shown in this study, has a significant impact on the community with a deterministic downturn in overall production.

### What Is the Limit of Reverse β-Oxidation Chain Elongation?

Octanoic acid has been previously detected in mixed community MCFA fermentations (Steinbusch et al., 2011; Van Eerten-Jansen et al., 2013; Spirito et al., 2014) but has not been conclusively identified as a product of reverse β-oxidation. This is partly due to the fact that it is not commonly expected as a likely product and therefore not necessarily included in analysis. Hexanoic acid production has been observed through reverse β-oxidation at up to 23.4 g L^−1^ (Zhu et al., 2015), which entertains the possibility that some of these microorganisms attempt to exact more energy by sending hexanoic acid through the reverse β-oxidation again. The fermentation by Zhu et al. (2015) was dominated by *Clostridium* group IV species engaged in lactic acid elongation, and it is not reported if the broth was tested for other MCFA. While reports of octanoic acid production are rare and exclusive to mixed culture fermentations, reports of decanoic acid as a microbial product are non-existent, to the author’s best knowledge. In this study, the cooccurrence of the C10 peaks with a high relative abundance of *Clostridium* sp. BS-1_sec_ and high concentrations of octanoic acid adds weight that decanoic acid may be an unreported reverse β-oxidation product that occurs once a threshold of octanoic acid has been passed, similar to the elongation of acetic acid to butyric, to hexanoic, and so on. The elongating species, in this case likely to be *Clostridium* sp. BS-1_sec_ (but perhaps also *Clostridium* sp. CPB-6), has outcompeted the flanking community to the point at which the available carbon and energy has becomes limited. One counter argument is the collapse of the flanking community, such as *Lactobacillus* spp., that coincides with the onset of the detection of octanoic acid or with sustained concentrations of hexanoic acid. Cellular fatty acid fragments from the dead cells may fit some of the correlations used here to support the C10 through reverse β-oxidation hypothesis, such as the increase in relative abundance of *Clostridium* sp. BS-1_sec_, which incounter can be viewed as the collapse in relative abundance of all other species. For example, the cellular fatty acid profile of *Lactobacillus* spp. can contain 2-hexyl-cyclopropanedecanoic acid (lactobacillic acid, C_19_H_36_O_2_), which contains a decanoic acid chain.

### Engineering and Understanding Complex, Mixed Culture MCFA Fermentation

Environmental factors such as temperature, pH, organic loading, and residence time, and to a certain extent, the mixed community inoculum, are under our control in an engineered system. In this study, all these factors were controlled in two identical reactors, chosen to be most conducive to the production of hexanoic acid, as is common practice in a mixed microbial community anaerobic MCFA fermentation. Though the two reactors had similar functional similarity, the community dissimilarity that arose led to *Clostridium* sp. BS-1_sec_ becoming the most relatively abundant microorganism, with octanoic and decanoic acid following and suppressing the activities of the rest of the community to the detriment of overall production. *Lactobacillus* spp. in particular decreased in relative abundance, which is likely to have resulted in a negative impact on conversion of the stillage into VFA. At the highest concentration of octanoic acid (day 39) in Reactor 1, the total VFA concentration was 59% of the total maximum VFA concentration (day 15). This phenomenon arose independently in the two identical, separate reactors operated in parallel, with the effect separated temporally by around 35 days. The highest concentration of hexanoic acid occurred in Reactor 2, and coincided with a minimum relative abundance of one of the likely producers of hexanoic acid, *Clostridium* sp. BS-1_sec_, during a period where *Clostridium* sp. BS-1_sec_, *Clostridium* sp. CPB-6, and *Lactobacillus* spp. each had a similar relative abundance in the vicinity of 15–40%. The greatest relative abundances of *Clostridium* sp. BS-1_sec_ coincided with a low hexanoic acid concentration and a low total VFA concentration. The system performed at its best—high conversion of substrate and a large proportion of hexanoic acid—when different species coexisted, while the presence of octanoic acid reduced the richness of the microbial community. Rather than optimizing reactor conditions to the benefit of *Clostridium* sp. BS-1_sec_, perhaps a better approach is ensuring a diverse biological economy to optimize anaerobic digestion and fermentation, alongside a targeted extraction of the problem compounds, if possible.

It is complicated to parse the origin of octanoic and decanoic acid, the rise in the relative abundance of *Clostridium* sp. BS-1_sec_, and the equal and opposite decrease in relative abundance of the other species, but the cooccurrence of *Clostridium* sp. BS-1_sec_ with the toxic octanoic acid product highlights the process risk of a winner-takes-all approach and encourages research into the interplay of the complex and competitive microbial community to engineer a stable, productive process that generate sustainable, valuable biochemicals. Toxicity in an MCFA fermentation, particularly in mixed cultures, has heretofore been viewed as the broad suppression of microorganisms leading to the collapse of production. In current literature, MCFA toxicity is only observed incidentally and, to our knowledge, has not been studied with a mixed culture. This study demonstrates varied tolerance to MCFA, and this variation within the community can lead to the dominance of some species while others succumb to the toxicity, which has a significant impact on the productivity of the fermentation.

## Author Contributions

SA designed and executed the experiments, analyzed the results, and wrote the manuscript. VD and WK executed some experiments and analyzed the results. RP and HR performed molecular analysis and designed and executed some statistical analyses. MC and KR contributed in design of experiments and interpretation of the results. All authors contributed to the final manuscript.

## Conflict of Interest Statement

The authors declare that the research was conducted in the absence of any commercial or financial relationships that could be construed as a potential conflict of interest.
