# Supplementary material for: A Clostridium Group IV Species Dominates and Suppresses a Mixed Culture Fermentation by Tolerance to Medium Chain Fatty Acids Products
Source: Front Bioeng Biotechnol. 2017 Feb 20;5:8. doi: 10.3389/fbioe.2017.00008 (PMC5316547; doi:10.3389/fbioe.2017.00008)
Supplement: Supplementary file 1 [file Data_Sheet_1.PDF]

Supplementary Material

A *Clostridium* Group IV species dominates and suppresses a mixed culture fermentation by tolerance to medium chain fatty acids products

Stephen J Andersen, Vicky De Groof, Way Cern Khor, Hugo Roume, Ruben Props, Marta Coma and Korneel Rabaey*

*** Correspondence:** Korneel Rabaey: korneel.rabaey@ugent.be

1 Supplementary Figures and Tables

1.1 Supplementary Tables

Table S1. Information for phylogenetic tree (Figure 2)

**Bacteria name**	**Sequence name**	**NCBI accession number**
Uncultured bacterium, clone: XZ10	gi|485461890|dbj|AB818631.1|	AB818631.1
Uncultured Clostridium sp. clone b2-173	gi|410699578|gb|JX576090.1|	JX576090.1
Uncultured bacterium clone 336 TC30 33	gi|669743720|gb|KM251001.1|	KM251001.1
Uncultured bacterium clone 161 TC18 65	gi|669743649|gb|KM250930.1|	KM250930.1
Uncultured bacterium clone T6_3_14	gi|212294855|gb|EU828413.1|	EU828413.1
Uncultured bacterium clone UASB_brew_B86	gi|13310399|gb|AF332721.1|	AF332721.1
Clostridium sp. BS-1	gi|330427205|gb|FJ805840.2|	FJ805840.2
Clostridium] sporosphaeroides strain ASW3.3	gi|891072069|gb|KT274733.1|	KT274733.1
Clostridium sp. CPB-6	gi|723265868|gb|KM454167.1|	KM454167.1
Ruminococcus bromii strain YE282	gi|113714178|gb|DQ882649.1|	DQ882649.1

Table S2.

Database: Nucleotide collection (nr/nt)

Exclude: Uncultured/environmental sample sequences

Program Selection: Highly similar sequences (megablast)

**Query**	**Subject**	**Max score**	**Total score**	**Query cover (%)**	**E value**	**Ident (%)**	**Accession**
OTU2	*Clostridium sp*. BS-1	628	628	100	2e-176	95	FJ805840.2
OTU4	*Clostridium sp*. BS-1	628	628	100	2e-176	95	FJ805840.2
OTU12	*Clostridium sp*. BS-1	595	595	100	2e-166	93	FJ805840.2
OTU19	*Clostridium sp*. BS-1	606	606	100	9e-170	94	FJ805840.2
OTU8	*Clostridium sp*. CPB-6	741	741	100	0.0	100	KM454167.1
OTU11	*Clostridium sp*. CPB-6	675	675	100	0.0	97	KM454167.1
OTU22	*Clostridium sp*. CPB-6	675	675	100	0.0	97	KM454167.1

Table S3.

Database: Nucleotide collection (nr/nt)

Exclude: Uncultured/environmental sample sequences

Program Selection: Highly similar sequences (megablast)

**Query**	**Subject**	**Max score**	**Total score**	**Query cover (%)**	**E value**	**Ident (%)**	**Accession**
Reactor-1 Day 34	Clostridium sp. BS-1	1587	1587	99	0.0	95	FJ805840.2
	Clostridium sp. CPB-6	1531	1531	99	0.0	94	KM454167.1

1.2 Supplementary Figures

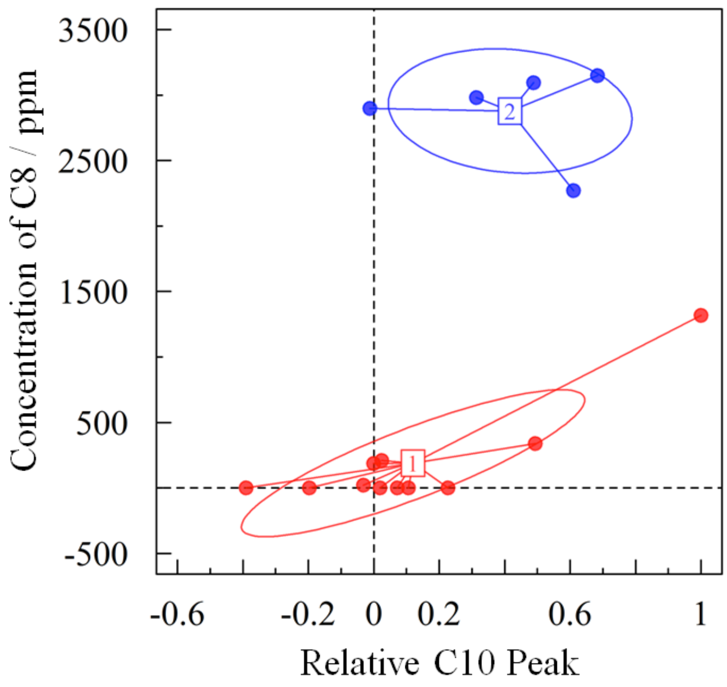

Figure S1. Relative decanoic acid (C10) peak plotted against the corresponding measured octanoic acid concentration, kmeans clustering with 2 centers for visualization purposes. (1) represents the negative detection cluster, while (2) represents the positive detection cluster. Note that a positive detection in (2) also requires a value in the Relative C10 Peak greater than 0.2 for that point to be considered a positive detection of C10.

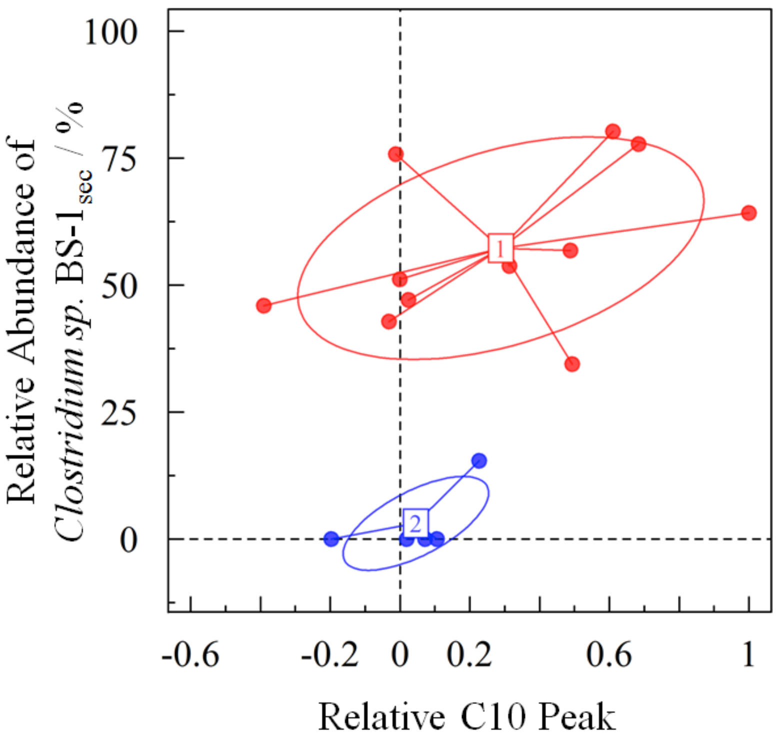

Figure S2. Relative decanoic acid (C10) peak plotted against the corresponding relative abundance of *Clostridium sp.* $BS1_{sec}$, kmeans clustering with 2 centres for visualization purposes. (2) represents the negative detection cluster, while (1) represents the positive detection cluster. Note that a positive detection in (1) also requires a value in the Relative C10 Peak greater than 0.2 for that point to be considered a detection of C10.

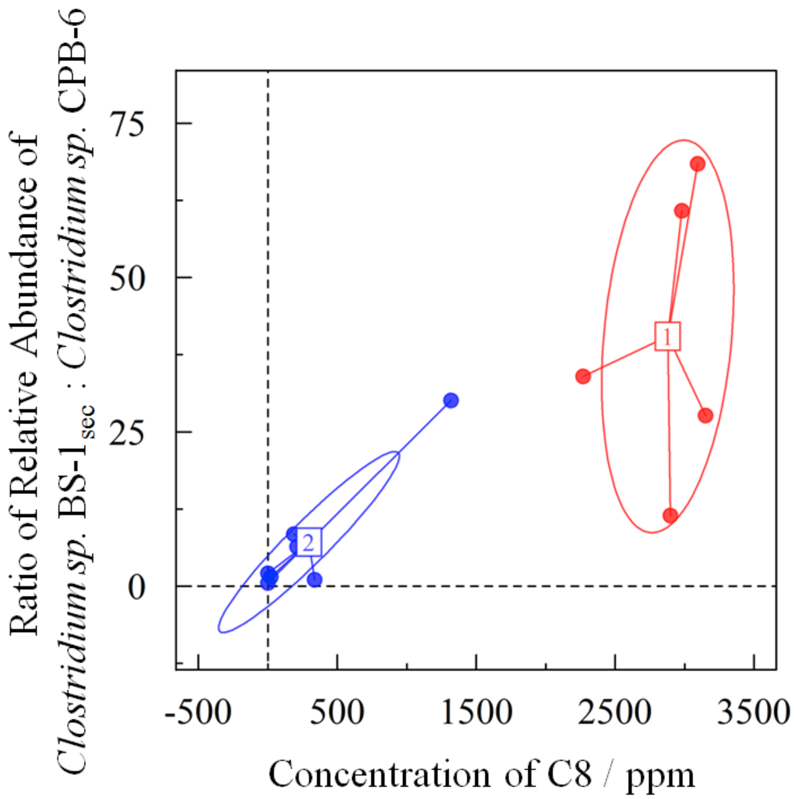

Figure S3. Concentration of C8 plotted against the corresponding ratio of the relative abundance of *Clostridium sp.* BS-1_{sec} to *Clostridium sp.* CPB-6, kmeans clustering with 2 centers for visualization purposes.

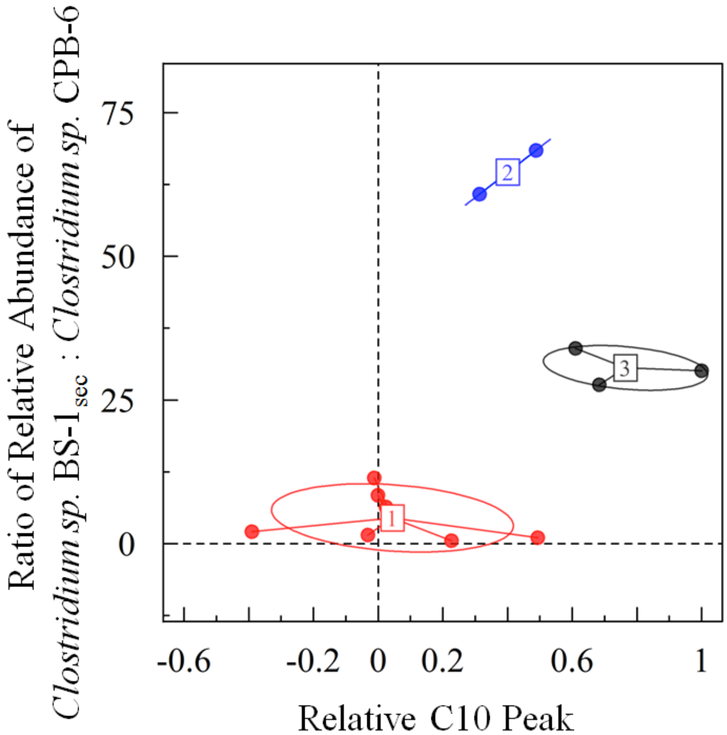

Figure S4. Relative C10 peak plotted against the corresponding ratio of the relative abundance of *Clostridium sp.* BS-1_{sec} to *Clostridium sp.* CPB-6, kmeans clustering with 3 centres for visualization purposes. (1) represents the negative detection cluster, while (2) and (3) represents the positive detection cluster. Note that a positive detection in (2) and (3) also requires a value in the Relative C10 Peak greater than 0.2 for that point to be considered a positive detection of C10.

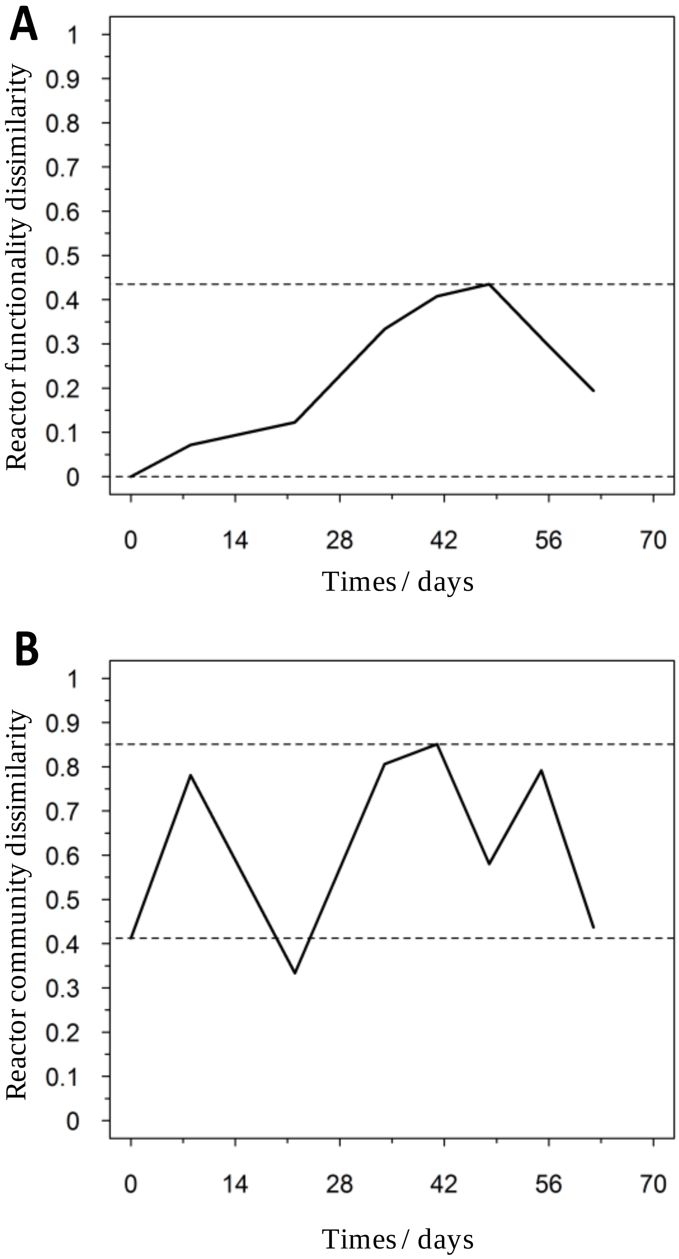

Figure S5. The level of dissimilarity between the reactors as measured in time (1 = dissimilar, 0 = identical). Dissimilarities between communities and functional profiles were calculated using the bray distance metric.
